# Bis(4,4′′-difluoro-1,1′:3′,1′′-terphenyl-2′-carboxyl­ato-κ*O*)bis­(3,5-dimethyl-1*H*-pyrazole-κ*N* ^2^)manganese(II)

**DOI:** 10.1107/S1600536812014201

**Published:** 2012-04-13

**Authors:** Sivanesan Dharmalingam, Yeojin Jeon, Sungho Yoon

**Affiliations:** a Department of Bio & Nano Chemistry, College of Natural Sciences, Kookmin University, 861-1 Jeongneung-dong, Seongbuk-gu, Seoul 136-702, Republic of Korea

## Abstract

In the title compound, [Mn(C_19_H_11_F_2_O_2_)_2_(C_5_H_8_N_2_)_2_], the Mn^2+^ cation is coordinated by the N atoms of two 3,5-dimethyl­pyrazole ligands and carboxyl­ate O atoms from two 4,4′′-difluoro-1,1′:3′,1′′-terphenyl-2′-carboxyl­ato ligands, forming an MnN_2_O_2_ polyhedron with a slightly distorted tetra­hedral coordination geometry. Two intra­molecular hydrogen bonds are observed between the carboxyl­ate and pyrazole ligands. The combined influence of the sterically hindered carboxyl­ate ligands and the intra­molecular hydrogen-bonding inter­actions stabilizes the title compound with a low coordination number of four. In the crystal, weak C—H⋯F and C—H⋯O hydrogen bonds are observed.

## Related literature

For the synthesis of substituted terphenyl-based carboxyl­ate ligands, see: Saednya & Hart (1996 ▶); Du *et al.* (1986 ▶); Chen & Siegel (1994 ▶). For background to metal complexes with terphenyl-based carboxyl­ate ligands, see: Kannan *et al.* (2011 ▶); Yoon & Lippard (2004*a* ▶,*b* ▶); Lee & Lippard (1998 ▶, 2001 ▶, 2002 ▶) and for those with 3,5-dimethyl­pyrazole ligands, see: Zhang *et al.* (2007 ▶); Cheng *et al.* (1990 ▶).
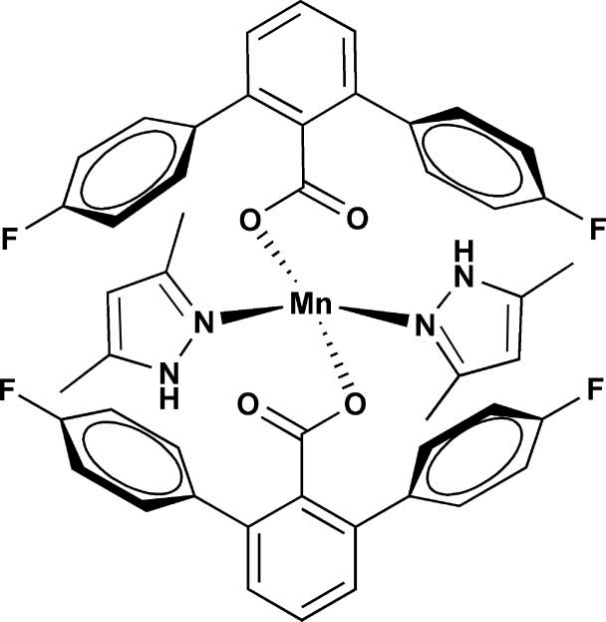


## Experimental

### 

#### Crystal data

[Mn(C_19_H_11_F_2_O_2_)_2_(C_5_H_8_N_2_)_2_]
*M* *_r_* = 865.76
Triclinic, 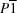

*a* = 10.9310 (16) Å
*b* = 13.668 (2) Å
*c* = 15.541 (2) Å
α = 69.283 (2)°
β = 88.854 (2)°
γ = 77.476 (2)°
*V* = 2115.9 (5) Å^3^
*Z* = 2
Mo *K*α radiation
μ = 0.38 mm^−1^
*T* = 173 K
0.10 × 0.10 × 0.05 mm

#### Data collection

Bruker SMART CCD area-detector diffractometer
Absorption correction: multi-scan (*SADABS*; Bruker, 2000 ▶) *T*_min_ = 0.469, *T*_max_ = 1.0
15553 measured reflections
7378 independent reflections
6578 reflections with *I* > 2σ(*I*)
*R*_int_ = 0.032

#### Refinement

*R*[*F* ^2^ > 2σ(*F* ^2^)] = 0.047
*wR*(*F* ^2^) = 0.118
*S* = 1.08
7378 reflections
562 parameters
H atoms treated by a mixture of independent and constrained refinement
Δρ_max_ = 0.67 e Å^−3^
Δρ_min_ = −0.37 e Å^−3^

### 

Data collection: *SMART* (Bruker, 2000 ▶); cell refinement: *SAINT* (Bruker, 2000 ▶); data reduction: *SAINT*; program(s) used to solve structure: *SHELXS97* (Sheldrick, 2008 ▶); program(s) used to refine structure: *SHELXL97* (Sheldrick, 2008 ▶); molecular graphics: *SHELXTL* (Sheldrick, 2008 ▶); software used to prepare material for publication: *SHELXTL*.

## Supplementary Material

Crystal structure: contains datablock(s) I, global. DOI: 10.1107/S1600536812014201/sj5226sup1.cif

Structure factors: contains datablock(s) I. DOI: 10.1107/S1600536812014201/sj5226Isup2.hkl

Additional supplementary materials:  crystallographic information; 3D view; checkCIF report

## Figures and Tables

**Table:** 

Mn1—O3	2.0636 (15)
Mn1—O1	2.0805 (16)
Mn1—N1	2.1292 (19)
Mn1—N3	2.1591 (19)

**Table:** 

O3—Mn1—O1	107.39 (7)
O3—Mn1—N1	105.08 (7)
O1—Mn1—N1	105.31 (7)
O3—Mn1—N3	101.78 (7)
O1—Mn1—N3	123.66 (7)
N1—Mn1—N3	112.13 (7)

**Table 2:** Hydrogen-bond geometry (Å, °)

*D*—H⋯*A*	*D*—H	H⋯*A*	*D*⋯*A*	*D*—H⋯*A*
N4—H2⋯O2	0.82 (3)	2.03 (3)	2.712 (3)	140 (3)
N2—H1⋯O4	0.87 (3)	1.95 (3)	2.783 (3)	160 (3)
C26—H20⋯F2^i^	0.95	2.56	3.355 (3)	141
C15—H16⋯O4^ii^	0.95	2.50	3.298 (3)	141
C38—H33⋯O3^iii^	0.95	2.70	3.606 (3)	160
C20—H23⋯F4^iv^	0.95	2.64	3.239 (3)	121
C45—H30⋯F3^v^	0.95	2.67	3.560 (4)	156

Symmetry codes: (i) 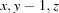; (ii) 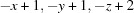; (iii) 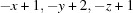; (iv) 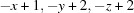; (v) 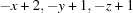.

**Fig. 1.**
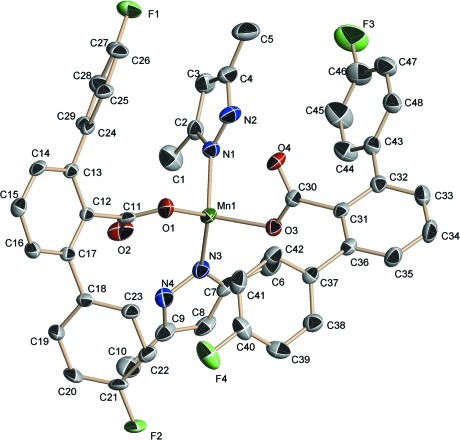
The molecular structure of the title compound, showing the atom-numbering and with displacement ellipsoids drawn at the 50% probability level. Hydrogen atoms are omitted for the clarity.

## supplementary crystallographic information

### Comment

4,4''-difluoro-[1,1':3',1''-terphenyl]-2'-carboxylato coordinated Fe^2+^ complexes are well known for their reactivity with dioxygen (Yoon & Lippard, 2004*a*,*b*; Lee & Lippard, 1998, 2001, 2002). The synthesis of terphenyl-based carboxylate ligands has been reported (Saednya *et al.*, 1996; Du *et al.*, 1986; Chen *et al.*, 1994). Also four coordinate Fe^2+^, Co^2+^, and Ni^2+^ metal complexes with a slightly distorted tetrahedral coordination geometry have also been reported with two 3,5-dimethylpyrazole and two 4,4''-difluoro-[1,1':3',1''-terphenyl]-2'-carboxylato ligands (Kannan *et al.* 2011; Yoon & Lippard, 2004*a*). Complexes with 3,5-dimethylpyrazole ligands have also been reported (Zhang *et al.*, 2007; Cheng *et al.*, 1990).

Here, we report the structure of a tetrahedrally coordinated Mn^2+^ complex which crystallizes in the triclinic space group P -1. Bond distances and bond angles to the metal are given in Table 1 with the structure of the molecule shown in Fig 1. In the crystal structure, weak intermolecular C—H···F and C—H···O hydrogen bonds, Table 2, stabilise the packing.

### Experimental

A portion of sodium [4,4''-difluoro-[1,1':3',1''-terphenyl]-2'-carboxylate] (0.110 g, 0.331 mmol) was mixed with Mn(OTf)_2_.2CH_3_CN (0.0720 g, 0.165 mmol) in 10 mL of tetrahydrofuran at room temperature. After 6 hours stirring, 3,5-dimethylpyrazole (0.0317g, 0.331 mmol) was added. After a further three hours, the tetrahydrofuran was removed under reduced pressure and colorless block-like crystals were collected using a dichloromethane and pentane layering system. Yield = 89%, (0.1268 g).

### Refinement

Hydrogen atoms bound to N were located in the difference Fourier map and refined isotropically. Other H atoms were placed in calculated positions and refined as riding with C—H (aromatic) = 0.95 Å, C—H(CH_3_) = 0.98 Å with [*U*iso(H) = 1.2 (1.5 for CH_3_ groups) *U*eq(C)].

### Crystal data

**Table:**

[Mn(C_19_H_11_F_2_O_2_)_2_(C_5_H_8_N_2_)_2_]	*Z* = 2
*M**_r_* = 865.76	*F*(000) = 894
Triclinic, *P*1	*D*_x_ = 1.359 Mg m^−^^3^
Hall symbol: -P 1	Mo *K*α radiation, λ = 0.71073 Å
*a* = 10.9310 (16) Å	Cell parameters from 1018 reflections
*b* = 13.668 (2) Å	θ = 2.5–27.2°
*c* = 15.541 (2) Å	µ = 0.38 mm^−^^1^
α = 69.283 (2)°	*T* = 173 K
β = 88.854 (2)°	Block, colorless
γ = 77.476 (2)°	0.10 × 0.10 × 0.05 mm
*V* = 2115.9 (5) Å^3^	

### Data collection

**Table:**

Bruker SMART CCD area-detector diffractometer	7378 independent reflections
Radiation source: fine-focus sealed tube	6578 reflections with *I* > 2σ(*I*)
Graphite monochromator	*R*_int_ = 0.032
φ and ω scans	θ_max_ = 25.0°, θ_min_ = 1.4°
Absorption correction: multi-scan (*SADABS*; Bruker, 2000)	*h* = −12→12
*T*_min_ = 0.469, *T*_max_ = 1.0	*k* = −16→16
15553 measured reflections	*l* = −18→18

### Refinement

**Table:**

Refinement on *F*^2^	Primary atom site location: structure-invariant direct methods
Least-squares matrix: full	Secondary atom site location: difference Fourier map
*R*[*F*^2^ > 2σ(*F*^2^)] = 0.047	Hydrogen site location: inferred from neighbouring sites
*wR*(*F*^2^) = 0.118	H atoms treated by a mixture of independent and constrained refinement
*S* = 1.08	*w* = 1/[σ^2^(*F*_o_^2^) + (0.0504*P*)^2^ + 1.1718*P*] where *P* = (*F*_o_^2^ + 2*F*_c_^2^)/3
7378 reflections	(Δ/σ)_max_ = 0.001
562 parameters	Δρ_max_ = 0.67 e Å^−^^3^
0 restraints	Δρ_min_ = −0.37 e Å^−^^3^

### Special details

**Table:**

Geometry. All esds (except the esd in the dihedral angle between two l.s. planes) are estimated using the full covariance matrix. The cell esds are taken into account individually in the estimation of esds in distances, angles and torsion angles; correlations between esds in cell parameters are only used when they are defined by crystal symmetry. An approximate (isotropic) treatment of cell esds is used for estimating esds involving l.s. planes.
Refinement. Refinement of F^2^ against ALL reflections. The weighted R-factor wR and goodness of fit S are based on F^2^, conventional R-factors R are based on F, with F set to zero for negative F^2^. The threshold expression of F^2^ > 2sigma(F^2^) is used only for calculating R-factors(gt) etc. and is not relevant to the choice of reflections for refinement. R-factors based on F^2^ are statistically about twice as large as those based on F, and R- factors based on ALL data will be even larger.

### Fractional atomic coordinates and isotropic or equivalent isotropic displacement parameters (Å^2^)

**Table:**

	*x*	*y*	*z*	*U*_iso_*/*U*_eq_	
C1	1.0866 (2)	0.5295 (3)	0.7746 (2)	0.0513 (8)	
H1A	1.1375	0.5522	0.7216	0.077*	
H2B	1.1399	0.4742	0.8266	0.077*	
H3C	1.0490	0.5913	0.7918	0.077*	
C2	0.9853 (2)	0.4851 (2)	0.74983 (16)	0.0314 (5)	
C3	0.9922 (3)	0.3873 (2)	0.73963 (18)	0.0407 (6)	
H3	1.0658	0.3327	0.7462	0.049*	
C4	0.8721 (3)	0.3847 (2)	0.71824 (18)	0.0422 (6)	
C5	0.8188 (4)	0.3015 (2)	0.7006 (3)	0.0709 (11)	
H5A	0.7848	0.2595	0.7571	0.106*	
H6B	0.8851	0.2538	0.6816	0.106*	
H7C	0.7516	0.3364	0.6517	0.106*	
C6	0.9539 (3)	0.8351 (2)	0.56558 (18)	0.0461 (7)	
H8A	0.9306	0.7668	0.5755	0.069*	
H9B	1.0398	0.8313	0.5455	0.069*	
H10C	0.8960	0.8932	0.5181	0.069*	
C7	0.9467 (2)	0.85669 (19)	0.65348 (16)	0.0314 (5)	
C8	0.9967 (2)	0.9287 (2)	0.67876 (18)	0.0376 (6)	
H4	1.0445	0.9768	0.6419	0.045*	
C9	0.9638 (2)	0.91730 (19)	0.76706 (17)	0.0338 (6)	
C10	0.9886 (3)	0.9709 (2)	0.8309 (2)	0.0507 (7)	
H12A	0.9175	0.9758	0.8697	0.076*	
H13B	0.9997	1.0432	0.7951	0.076*	
H14C	1.0650	0.9291	0.8699	0.076*	
C11	0.7265 (2)	0.64209 (16)	0.92263 (15)	0.0249 (5)	
C12	0.67639 (19)	0.60871 (16)	1.01669 (14)	0.0185 (4)	
C17	0.63182 (19)	0.68616 (17)	1.05611 (14)	0.0212 (4)	
C16	0.5773 (2)	0.65568 (18)	1.14060 (14)	0.0264 (5)	
H15	0.5498	0.7069	1.1693	0.032*	
C15	0.5629 (2)	0.55161 (19)	1.18310 (15)	0.0284 (5)	
H16	0.5220	0.5325	1.2392	0.034*	
C14	0.6080 (2)	0.47540 (17)	1.14401 (15)	0.0260 (5)	
H17	0.5974	0.4042	1.1735	0.031*	
C13	0.66876 (19)	0.50138 (17)	1.06224 (14)	0.0211 (4)	
C24	0.7289 (2)	0.41609 (16)	1.02611 (14)	0.0223 (5)	
C29	0.8558 (2)	0.40187 (18)	1.00785 (16)	0.0278 (5)	
H18	0.9031	0.4487	1.0165	0.033*	
C28	0.9137 (2)	0.3206 (2)	0.97736 (17)	0.0342 (6)	
H19	1.0001	0.3110	0.9652	0.041*	
C27	0.8434 (3)	0.25472 (19)	0.96519 (17)	0.0359 (6)	
C26	0.7184 (2)	0.26476 (19)	0.98273 (18)	0.0373 (6)	
H20	0.6723	0.2173	0.9739	0.045*	
C25	0.6619 (2)	0.34618 (17)	1.01366 (16)	0.0298 (5)	
H21	0.5757	0.3543	1.0266	0.036*	
C18	0.6370 (2)	0.80062 (17)	1.00680 (14)	0.0223 (5)	
C19	0.7005 (3)	0.85289 (19)	1.04621 (16)	0.0338 (6)	
H22	0.7408	0.8157	1.1061	0.041*	
C20	0.7060 (3)	0.9594 (2)	0.99926 (18)	0.0407 (6)	
H23	0.7502	0.9953	1.0261	0.049*	
C21	0.6463 (2)	1.01103 (17)	0.91372 (16)	0.0306 (5)	
C22	0.5818 (2)	0.96331 (19)	0.87257 (16)	0.0318 (5)	
H24	0.5406	1.0018	0.8131	0.038*	
C23	0.5775 (2)	0.85704 (18)	0.91944 (16)	0.0282 (5)	
H25	0.5334	0.8222	0.8915	0.034*	
C30	0.57304 (19)	0.71049 (17)	0.60758 (14)	0.0232 (5)	
C31	0.4892 (2)	0.77015 (17)	0.52004 (14)	0.0227 (5)	
C36	0.3932 (2)	0.85918 (18)	0.51369 (15)	0.0263 (5)	
C35	0.3179 (2)	0.9123 (2)	0.43246 (16)	0.0361 (6)	
H26	0.2527	0.9729	0.4278	0.043*	
C34	0.3364 (3)	0.8786 (2)	0.35867 (17)	0.0406 (6)	
H27	0.2837	0.9153	0.3038	0.049*	
C33	0.4312 (2)	0.7916 (2)	0.36461 (16)	0.0349 (6)	
H28	0.4444	0.7694	0.3131	0.042*	
C32	0.5081 (2)	0.73568 (18)	0.44486 (15)	0.0263 (5)	
C43	0.6113 (2)	0.64322 (19)	0.44768 (15)	0.0286 (5)	
C44	0.7327 (2)	0.6363 (2)	0.47775 (19)	0.0412 (6)	
H29	0.7506	0.6921	0.4950	0.049*	
C45	0.8276 (3)	0.5491 (3)	0.4828 (2)	0.0598 (9)	
H30	0.9107	0.5440	0.5037	0.072*	
C46	0.7997 (3)	0.4703 (3)	0.4571 (2)	0.0541 (8)	
C47	0.6825 (3)	0.4741 (2)	0.42535 (19)	0.0458 (7)	
H31	0.6663	0.4184	0.4072	0.055*	
C48	0.5882 (2)	0.5621 (2)	0.42059 (16)	0.0348 (6)	
H32	0.5059	0.5671	0.3983	0.042*	
C37	0.3671 (2)	0.89838 (18)	0.59182 (15)	0.0251 (5)	
C38	0.3556 (2)	1.0065 (2)	0.57712 (17)	0.0343 (6)	
H33	0.3666	1.0546	0.5176	0.041*	
C39	0.3282 (2)	1.0448 (2)	0.64837 (19)	0.0406 (6)	
H34	0.3214	1.1184	0.6387	0.049*	
C40	0.3111 (2)	0.9740 (2)	0.73290 (18)	0.0378 (6)	
C41	0.3204 (2)	0.8674 (2)	0.75057 (17)	0.0387 (6)	
H35	0.3076	0.8204	0.8101	0.046*	
C42	0.3490 (2)	0.8299 (2)	0.67922 (16)	0.0321 (5)	
H36	0.3563	0.7559	0.6901	0.039*	
F1	0.90001 (16)	0.17383 (12)	0.93543 (12)	0.0536 (4)	
F2	0.65221 (16)	1.11511 (11)	0.86681 (10)	0.0464 (4)	
F3	0.89370 (19)	0.38355 (17)	0.46375 (17)	0.0866 (7)	
F4	0.28254 (16)	1.01216 (16)	0.80295 (12)	0.0579 (5)	
Mn1	0.76676 (3)	0.68954 (3)	0.74376 (2)	0.02314 (11)	
N1	0.86659 (17)	0.54142 (15)	0.73521 (13)	0.0271 (4)	
N2	0.7995 (2)	0.47746 (16)	0.71690 (15)	0.0362 (5)	
N3	0.88491 (18)	0.80274 (15)	0.72287 (13)	0.0286 (4)	
N4	0.89665 (19)	0.84149 (17)	0.79094 (15)	0.0324 (5)	
O1	0.65725 (16)	0.64746 (13)	0.85637 (10)	0.0349 (4)	
O2	0.83122 (17)	0.66487 (14)	0.91176 (13)	0.0421 (5)	
O3	0.64655 (14)	0.76094 (12)	0.62699 (10)	0.0274 (4)	
O4	0.56667 (15)	0.61680 (12)	0.65369 (11)	0.0307 (4)	
H1	0.721 (3)	0.507 (2)	0.699 (2)	0.045 (8)*	
H2	0.875 (3)	0.808 (2)	0.842 (2)	0.039 (8)*	

### Atomic displacement parameters (Å^2^)

**Table:**

	*U*^11^	*U*^22^	*U*^33^	*U*^12^	*U*^13^	*U*^23^
C1	0.0296 (14)	0.064 (2)	0.0631 (19)	−0.0012 (13)	−0.0081 (13)	−0.0312 (16)
C2	0.0282 (12)	0.0352 (14)	0.0268 (12)	0.0010 (10)	−0.0019 (10)	−0.0108 (10)
C3	0.0394 (14)	0.0352 (15)	0.0405 (14)	0.0093 (12)	−0.0051 (12)	−0.0151 (12)
C4	0.0536 (17)	0.0254 (14)	0.0418 (15)	−0.0035 (12)	−0.0111 (13)	−0.0074 (11)
C5	0.092 (3)	0.0313 (17)	0.086 (3)	−0.0122 (17)	−0.025 (2)	−0.0165 (17)
C6	0.0505 (16)	0.0426 (16)	0.0366 (14)	−0.0090 (13)	0.0153 (13)	−0.0054 (12)
C7	0.0254 (12)	0.0264 (12)	0.0298 (12)	−0.0028 (10)	0.0039 (10)	0.0030 (10)
C8	0.0332 (13)	0.0286 (13)	0.0403 (14)	−0.0142 (11)	0.0042 (11)	0.0046 (11)
C9	0.0278 (12)	0.0278 (13)	0.0391 (14)	−0.0094 (10)	−0.0036 (11)	−0.0018 (11)
C10	0.0547 (18)	0.0441 (17)	0.0580 (18)	−0.0266 (14)	−0.0036 (14)	−0.0147 (14)
C11	0.0342 (13)	0.0116 (10)	0.0271 (12)	−0.0057 (9)	0.0090 (10)	−0.0049 (9)
C12	0.0201 (10)	0.0164 (10)	0.0197 (10)	−0.0088 (8)	0.0003 (8)	−0.0043 (8)
C17	0.0238 (11)	0.0188 (11)	0.0219 (10)	−0.0081 (9)	0.0006 (9)	−0.0063 (9)
C16	0.0318 (12)	0.0263 (12)	0.0223 (11)	−0.0076 (10)	0.0041 (9)	−0.0099 (9)
C15	0.0324 (12)	0.0302 (13)	0.0196 (11)	−0.0104 (10)	0.0071 (9)	−0.0035 (9)
C14	0.0300 (12)	0.0181 (11)	0.0249 (11)	−0.0101 (9)	0.0006 (9)	0.0011 (9)
C13	0.0210 (10)	0.0190 (11)	0.0223 (10)	−0.0084 (9)	−0.0010 (9)	−0.0035 (9)
C24	0.0303 (12)	0.0144 (10)	0.0191 (10)	−0.0074 (9)	−0.0020 (9)	−0.0010 (8)
C29	0.0332 (12)	0.0225 (12)	0.0312 (12)	−0.0128 (10)	0.0045 (10)	−0.0100 (10)
C28	0.0362 (13)	0.0310 (13)	0.0347 (13)	−0.0053 (11)	0.0054 (11)	−0.0122 (11)
C27	0.0523 (16)	0.0206 (12)	0.0327 (13)	0.0016 (11)	−0.0031 (12)	−0.0123 (10)
C26	0.0471 (15)	0.0206 (12)	0.0468 (15)	−0.0092 (11)	−0.0104 (12)	−0.0135 (11)
C25	0.0318 (12)	0.0186 (11)	0.0368 (13)	−0.0077 (10)	−0.0056 (10)	−0.0058 (10)
C18	0.0264 (11)	0.0176 (11)	0.0243 (11)	−0.0057 (9)	0.0068 (9)	−0.0091 (9)
C19	0.0551 (16)	0.0243 (12)	0.0246 (11)	−0.0147 (11)	−0.0021 (11)	−0.0080 (10)
C20	0.0689 (19)	0.0279 (13)	0.0364 (14)	−0.0245 (13)	−0.0007 (13)	−0.0166 (11)
C21	0.0475 (15)	0.0136 (11)	0.0325 (12)	−0.0099 (10)	0.0093 (11)	−0.0089 (10)
C22	0.0367 (13)	0.0224 (12)	0.0309 (12)	−0.0065 (10)	−0.0016 (10)	−0.0031 (10)
C23	0.0314 (12)	0.0210 (12)	0.0329 (12)	−0.0105 (10)	−0.0016 (10)	−0.0077 (10)
C30	0.0205 (10)	0.0248 (12)	0.0216 (11)	−0.0022 (9)	0.0043 (9)	−0.0069 (9)
C31	0.0240 (11)	0.0200 (11)	0.0213 (10)	−0.0084 (9)	0.0024 (9)	−0.0023 (9)
C36	0.0283 (12)	0.0229 (12)	0.0229 (11)	−0.0065 (9)	0.0015 (9)	−0.0021 (9)
C35	0.0368 (14)	0.0308 (14)	0.0304 (13)	0.0042 (11)	−0.0035 (11)	−0.0052 (11)
C34	0.0454 (15)	0.0421 (16)	0.0240 (12)	0.0006 (12)	−0.0097 (11)	−0.0050 (11)
C33	0.0423 (14)	0.0397 (15)	0.0228 (11)	−0.0099 (12)	0.0010 (10)	−0.0110 (11)
C32	0.0287 (12)	0.0260 (12)	0.0236 (11)	−0.0107 (10)	0.0036 (9)	−0.0057 (9)
C43	0.0314 (12)	0.0315 (13)	0.0232 (11)	−0.0097 (10)	0.0075 (9)	−0.0087 (10)
C44	0.0329 (14)	0.0460 (16)	0.0538 (16)	−0.0088 (12)	0.0096 (12)	−0.0291 (14)
C45	0.0311 (14)	0.076 (2)	0.082 (2)	0.0030 (15)	0.0018 (15)	−0.049 (2)
C46	0.0492 (18)	0.0503 (18)	0.0619 (19)	0.0108 (14)	0.0078 (15)	−0.0324 (16)
C47	0.0600 (18)	0.0408 (16)	0.0425 (15)	−0.0116 (14)	0.0129 (14)	−0.0223 (13)
C48	0.0396 (14)	0.0377 (14)	0.0297 (12)	−0.0099 (12)	0.0076 (11)	−0.0147 (11)
C37	0.0213 (11)	0.0246 (12)	0.0257 (11)	−0.0025 (9)	0.0006 (9)	−0.0060 (9)
C38	0.0378 (14)	0.0293 (13)	0.0349 (13)	−0.0113 (11)	0.0059 (11)	−0.0083 (11)
C39	0.0427 (15)	0.0352 (15)	0.0531 (16)	−0.0162 (12)	0.0076 (13)	−0.0227 (13)
C40	0.0298 (13)	0.0550 (17)	0.0383 (14)	−0.0087 (12)	0.0034 (11)	−0.0287 (13)
C41	0.0371 (14)	0.0458 (16)	0.0248 (12)	−0.0029 (12)	0.0042 (11)	−0.0062 (11)
C42	0.0330 (13)	0.0264 (13)	0.0293 (12)	−0.0015 (10)	0.0022 (10)	−0.0038 (10)
F1	0.0702 (11)	0.0338 (9)	0.0608 (10)	0.0030 (8)	−0.0018 (9)	−0.0298 (8)
F2	0.0813 (12)	0.0172 (7)	0.0421 (8)	−0.0208 (7)	0.0021 (8)	−0.0067 (6)
F3	0.0693 (13)	0.0766 (14)	0.1162 (18)	0.0258 (11)	−0.0018 (12)	−0.0617 (14)
F4	0.0544 (10)	0.0866 (13)	0.0533 (10)	−0.0181 (9)	0.0114 (8)	−0.0488 (10)
Mn1	0.02515 (19)	0.01969 (19)	0.02176 (18)	−0.00732 (14)	0.00321 (13)	−0.00279 (14)
N1	0.0261 (10)	0.0254 (10)	0.0291 (10)	−0.0053 (8)	0.0003 (8)	−0.0094 (8)
N2	0.0303 (11)	0.0240 (11)	0.0487 (13)	−0.0055 (9)	−0.0096 (10)	−0.0062 (10)
N3	0.0305 (10)	0.0273 (11)	0.0254 (10)	−0.0126 (9)	0.0033 (8)	−0.0030 (8)
N4	0.0365 (11)	0.0351 (12)	0.0279 (11)	−0.0208 (10)	0.0053 (9)	−0.0068 (9)
O1	0.0490 (10)	0.0341 (10)	0.0195 (8)	−0.0078 (8)	0.0050 (7)	−0.0081 (7)
O2	0.0407 (10)	0.0342 (10)	0.0510 (11)	−0.0208 (8)	0.0231 (9)	−0.0086 (9)
O3	0.0287 (8)	0.0275 (9)	0.0252 (8)	−0.0083 (7)	−0.0018 (7)	−0.0071 (7)
O4	0.0306 (9)	0.0218 (8)	0.0299 (8)	−0.0063 (7)	0.0002 (7)	0.0029 (7)

### Geometric parameters (Å, º)

**Table:**

C1—C2	1.489 (4)	C19—C20	1.393 (3)
C1—H1A	0.9800	C19—H22	0.9500
C1—H2B	0.9800	C20—C21	1.365 (4)
C1—H3C	0.9800	C20—H23	0.9500
C2—N1	1.335 (3)	C21—C22	1.358 (3)
C2—C3	1.387 (4)	C21—F2	1.365 (3)
C3—C4	1.371 (4)	C22—C23	1.386 (3)
C3—H3	0.9500	C22—H24	0.9500
C4—N2	1.335 (3)	C23—H25	0.9500
C4—C5	1.491 (4)	C30—O4	1.243 (3)
C5—H5A	0.9800	C30—O3	1.269 (3)
C5—H6B	0.9800	C30—C31	1.516 (3)
C5—H7C	0.9800	C31—C36	1.399 (3)
C6—C7	1.493 (4)	C31—C32	1.403 (3)
C6—H8A	0.9800	C36—C35	1.390 (3)
C6—H9B	0.9800	C36—C37	1.491 (3)
C6—H10C	0.9800	C35—C34	1.376 (4)
C7—N3	1.337 (3)	C35—H26	0.9500
C7—C8	1.393 (4)	C34—C33	1.374 (4)
C8—C9	1.375 (4)	C34—H27	0.9500
C8—H4	0.9500	C33—C32	1.391 (3)
C9—N4	1.341 (3)	C33—H28	0.9500
C9—C10	1.485 (4)	C32—C43	1.488 (3)
C10—H12A	0.9800	C43—C48	1.387 (3)
C10—H13B	0.9800	C43—C44	1.389 (3)
C10—H14C	0.9800	C44—C45	1.379 (4)
C11—O2	1.243 (3)	C44—H29	0.9500
C11—O1	1.262 (3)	C45—C46	1.366 (4)
C11—C12	1.500 (3)	C45—H30	0.9500
C12—C17	1.399 (3)	C46—F3	1.365 (3)
C12—C13	1.406 (3)	C46—C47	1.366 (4)
C17—C16	1.393 (3)	C47—C48	1.385 (4)
C17—C18	1.492 (3)	C47—H31	0.9500
C16—C15	1.383 (3)	C48—H32	0.9500
C16—H15	0.9500	C37—C42	1.389 (3)
C15—C14	1.382 (3)	C37—C38	1.391 (3)
C15—H16	0.9500	C38—C39	1.385 (4)
C14—C13	1.390 (3)	C38—H33	0.9500
C14—H17	0.9500	C39—C40	1.366 (4)
C13—C24	1.491 (3)	C39—H34	0.9500
C24—C25	1.388 (3)	C40—C41	1.365 (4)
C24—C29	1.396 (3)	C40—F4	1.368 (3)
C29—C28	1.383 (3)	C41—C42	1.382 (3)
C29—H18	0.9500	C41—H35	0.9500
C28—C27	1.365 (4)	C42—H36	0.9500
C28—H19	0.9500	Mn1—O3	2.0636 (15)
C27—F1	1.368 (3)	Mn1—O1	2.0805 (16)
C27—C26	1.375 (4)	Mn1—N1	2.1292 (19)
C26—C25	1.386 (3)	Mn1—N3	2.1591 (19)
C26—H20	0.9500	N1—N2	1.358 (3)
C25—H21	0.9500	N2—H1	0.87 (3)
C18—C19	1.381 (3)	N3—N4	1.359 (3)
C18—C23	1.392 (3)	N4—H2	0.82 (3)
			
C2—C1—H1A	109.5	C21—C20—H23	120.8
C2—C1—H2B	109.5	C19—C20—H23	120.8
H1A—C1—H2B	109.5	C22—C21—C20	123.0 (2)
C2—C1—H3C	109.5	C22—C21—F2	118.3 (2)
H1A—C1—H3C	109.5	C20—C21—F2	118.7 (2)
H2B—C1—H3C	109.5	C21—C22—C23	118.3 (2)
N1—C2—C3	110.1 (2)	C21—C22—H24	120.8
N1—C2—C1	120.2 (2)	C23—C22—H24	120.8
C3—C2—C1	129.7 (2)	C22—C23—C18	121.0 (2)
C4—C3—C2	106.6 (2)	C22—C23—H25	119.5
C4—C3—H3	126.7	C18—C23—H25	119.5
C2—C3—H3	126.7	O4—C30—O3	125.1 (2)
N2—C4—C3	106.1 (2)	O4—C30—C31	118.58 (19)
N2—C4—C5	121.8 (3)	O3—C30—C31	116.30 (18)
C3—C4—C5	132.1 (3)	C36—C31—C32	119.83 (19)
C4—C5—H5A	109.5	C36—C31—C30	120.54 (19)
C4—C5—H6B	109.5	C32—C31—C30	119.63 (19)
H5A—C5—H6B	109.5	C35—C36—C31	119.2 (2)
C4—C5—H7C	109.5	C35—C36—C37	118.5 (2)
H5A—C5—H7C	109.5	C31—C36—C37	122.26 (19)
H6B—C5—H7C	109.5	C34—C35—C36	121.0 (2)
C7—C6—H8A	109.5	C34—C35—H26	119.5
C7—C6—H9B	109.5	C36—C35—H26	119.5
H8A—C6—H9B	109.5	C33—C34—C35	119.8 (2)
C7—C6—H10C	109.5	C33—C34—H27	120.1
H8A—C6—H10C	109.5	C35—C34—H27	120.1
H9B—C6—H10C	109.5	C34—C33—C32	121.0 (2)
N3—C7—C8	109.6 (2)	C34—C33—H28	119.5
N3—C7—C6	120.6 (2)	C32—C33—H28	119.5
C8—C7—C6	129.9 (2)	C33—C32—C31	119.1 (2)
C9—C8—C7	107.1 (2)	C33—C32—C43	119.2 (2)
C9—C8—H4	126.5	C31—C32—C43	121.63 (19)
C7—C8—H4	126.5	C48—C43—C44	118.6 (2)
N4—C9—C8	105.6 (2)	C48—C43—C32	120.8 (2)
N4—C9—C10	121.8 (2)	C44—C43—C32	120.6 (2)
C8—C9—C10	132.7 (2)	C45—C44—C43	120.6 (3)
C9—C10—H12A	109.5	C45—C44—H29	119.7
C9—C10—H13B	109.5	C43—C44—H29	119.7
H12A—C10—H13B	109.5	C46—C45—C44	118.6 (3)
C9—C10—H14C	109.5	C46—C45—H30	120.7
H12A—C10—H14C	109.5	C44—C45—H30	120.7
H13B—C10—H14C	109.5	F3—C46—C47	118.6 (3)
O2—C11—O1	122.4 (2)	F3—C46—C45	118.2 (3)
O2—C11—C12	120.4 (2)	C47—C46—C45	123.2 (3)
O1—C11—C12	117.16 (19)	C46—C47—C48	117.5 (3)
C17—C12—C13	120.69 (19)	C46—C47—H31	121.2
C17—C12—C11	119.38 (18)	C48—C47—H31	121.2
C13—C12—C11	119.85 (18)	C47—C48—C43	121.4 (2)
C16—C17—C12	118.90 (19)	C47—C48—H32	119.3
C16—C17—C18	120.29 (19)	C43—C48—H32	119.3
C12—C17—C18	120.74 (18)	C42—C37—C38	118.6 (2)
C15—C16—C17	120.6 (2)	C42—C37—C36	121.5 (2)
C15—C16—H15	119.7	C38—C37—C36	119.9 (2)
C17—C16—H15	119.7	C39—C38—C37	120.7 (2)
C14—C15—C16	120.0 (2)	C39—C38—H33	119.7
C14—C15—H16	120.0	C37—C38—H33	119.7
C16—C15—H16	120.0	C40—C39—C38	118.4 (2)
C15—C14—C13	121.0 (2)	C40—C39—H34	120.8
C15—C14—H17	119.5	C38—C39—H34	120.8
C13—C14—H17	119.5	C41—C40—C39	123.1 (2)
C14—C13—C12	118.6 (2)	C41—C40—F4	118.5 (2)
C14—C13—C24	120.40 (19)	C39—C40—F4	118.3 (3)
C12—C13—C24	121.00 (19)	C40—C41—C42	118.0 (2)
C25—C24—C29	118.5 (2)	C40—C41—H35	121.0
C25—C24—C13	121.0 (2)	C42—C41—H35	121.0
C29—C24—C13	120.49 (19)	C41—C42—C37	121.2 (2)
C28—C29—C24	121.0 (2)	C41—C42—H36	119.4
C28—C29—H18	119.5	C37—C42—H36	119.4
C24—C29—H18	119.5	O3—Mn1—O1	107.39 (7)
C27—C28—C29	118.3 (2)	O3—Mn1—N1	105.08 (7)
C27—C28—H19	120.9	O1—Mn1—N1	105.31 (7)
C29—C28—H19	120.9	O3—Mn1—N3	101.78 (7)
C28—C27—F1	118.7 (2)	O1—Mn1—N3	123.66 (7)
C28—C27—C26	123.0 (2)	N1—Mn1—N3	112.13 (7)
F1—C27—C26	118.2 (2)	C2—N1—N2	105.00 (19)
C27—C26—C25	118.0 (2)	C2—N1—Mn1	136.67 (16)
C27—C26—H20	121.0	N2—N1—Mn1	118.13 (14)
C25—C26—H20	121.0	C4—N2—N1	112.3 (2)
C26—C25—C24	121.1 (2)	C4—N2—H1	131.1 (19)
C26—C25—H21	119.4	N1—N2—H1	115.9 (19)
C24—C25—H21	119.4	C7—N3—N4	105.33 (19)
C19—C18—C23	118.6 (2)	C7—N3—Mn1	136.36 (17)
C19—C18—C17	121.18 (19)	N4—N3—Mn1	118.01 (14)
C23—C18—C17	120.26 (19)	C9—N4—N3	112.5 (2)
C18—C19—C20	120.8 (2)	C9—N4—H2	130 (2)
C18—C19—H22	119.6	N3—N4—H2	117 (2)
C20—C19—H22	119.6	C11—O1—Mn1	103.42 (14)
C21—C20—C19	118.3 (2)	C30—O3—Mn1	120.59 (14)

### Hydrogen-bond geometry (Å, º)

**Table:**

*D*—H···*A*	*D*—H	H···*A*	*D*···*A*	*D*—H···*A*
N4—H2···O2	0.82 (3)	2.03 (3)	2.712 (3)	140 (3)
N2—H1···O4	0.87 (3)	1.95 (3)	2.783 (3)	160 (3)
C26—H20···F2^i^	0.95	2.56	3.355 (3)	141
C15—H16···O4^ii^	0.95	2.50	3.298 (3)	141
C38—H33···O3^iii^	0.95	2.70	3.606 (3)	160
C20—H23···F4^iv^	0.95	2.64	3.239 (3)	121
C45—H30···F3^v^	0.95	2.67	3.560 (4)	156

Symmetry codes: (i) *x*, *y*−1, *z*; (ii) −*x*+1, −*y*+1, −*z*+2; (iii) −*x*+1, −*y*+2, −*z*+1; (iv) −*x*+1, −*y*+2, −*z*+2; (v) −*x*+2, −*y*+1, −*z*+1.
